# The interplay between cytokine genes and microRNAs in anemia of inflammation among hemodialysis patients

**DOI:** 10.1038/s41598-026-49829-w

**Published:** 2026-05-18

**Authors:** Mohamed Shemis, Omar M. Sabry, Nevine Sherif, Samah Mamdouh, Noha Elsheikh, Samia El-Shishtawy, Tamer Abdeltawab, Ola B. Abo El Nil, Amany M. Kamal, Sherihan G. AbdelHamid

**Affiliations:** 1 Biochemistry and Molecular Biology Department, Theodor Bilharz Research Institute, Giza, Egypt; 2 Hematology Department, Theodor Bilharz Research Institute, Giza, Egypt; 3 Nephrology Department, Theodor Bilharz Research Institute, Giza, Egypt; 4 Biochemistry and Molecular Biology Department, Faculty of Pharmacy, Ain Shams University, African Union Organization Street, Cairo, 11566 Egypt

**Keywords:** Anemia of inflammation, Hemodialysis, IL-6, TNF-α, MiR-34, MiR-130, MiR-16b, Biomarkers, Computational biology and bioinformatics, Diseases, Immunology, Nephrology

## Abstract

Anemia of inflammation (AI) represents the second most prevalent anemia globally particularly in conditions of prolonged immune activation including hemodialysis (HD). While the role of pro-inflammatory cytokines in disrupting iron homeostasis and erythropoiesis is well-established, the regulatory mechanisms involving microRNAs (miRNAs) remain incompletely elucidated. The current study investigates the intricate relationships between cytokines and miRNAs in the pathogenesis of AI among HD patients. The study comprised 30 HD patients with AI and 30 healthy controls. Expression profile of inflammatory cytokines (IL-6, TNF-α) and inflammation-associated miRNAs (miR-34, miR-130, miR-16b) were analyzed using quantitative real-time PCR. Serum C-reactive protein (CRP) and iron metabolism markers (serum iron, ferritin, transferrin saturation and total iron binding capacity) were analyzed. Correlation analyses and pathway enrichment studies were performed to identify cytokine-miRNA regulatory networks. HD with AI exhibited significant overexpression of IL-6, TNF-α and miR-34 (*p* < 0.001), while miR-130 and miR-16b were significantly downregulated (*P* < 0.001) compared to control group. MiR-34 was positively correlated with IL-6 (*r* = 0.96, *p* = 0.001) and TNF-α (*r* = 0.98, *p =* 0.001), while it showed significant inverse correlation with miR-130 and miR-16b (*p =* 0.001 for both). Furthermore, strong positive associations were observed between lower levels of transferrin saturation and decreased expression of IL-6, TNF-α and miR-16b (*p =* 0.01, 0.009, 0.001 respectively). The markedly increased ferritin levels (> 600 mg/mL) showed direct positive relationship with elevated expression of IL-6 and TNF-α in HD with AI. The present findings are exploratory and hypothesis-generating, suggesting possible relationship between miRNA dysregulation and the development of AI in HD patients, and provide the rationale for external validation in larger, independent HD cohorts. The interaction between miRNA expression and cytokine signaling may provide novel insights into the mechanism that could perpetuate AI in HD unravelling potential therapeutic targets that could mitigate the inflammatory response ultimately improving patient outcomes.

**Supplementary Information:**

The online version contains supplementary material available at 10.1038/s41598-026-49829-w.

## Introduction

Anemia of inflammation (AI), also known as anemia of chronic disease, is the second most prevalent anemia worldwide^1^. AI is defined as moderate to moderately severe anemia that occurs in cases of systemic inflammation due to decreased erythrocyte production and survival^2,3^. Similar to iron deficiency anemia (IDA), it is characterized by low serum iron, but in contrast, the iron storage is maintained in the reticuloendothelial system (RES), which recycles the erythrocytes^3,4^. AI is mainly a disorder of iron distribution caused by macrophage activation syndrome and occurs particularly in conditions of prolonged immune activation including hemodialysis (HD)^1,4^. In cases of systemic inflammation, as evidenced by elevated levels of C-reactive protein (CRP), WHO defines iron deficiency as ferritin concentration less than 70 µg/L in adults^5,6^.

AI affects approximately 80–90% of maintenance HD patients and represents a significant leading cause of morbidity, reduced quality of life and mortality in chronic kidney disease (CKD)^4^. HD accounts for approximately 69% of all renal replacement therapy, with a number of patients exceeding 2.5 million worldwide in 2020 and is expected to reach 5.4 million in 2030^7–9^. HD patients represent a unique population for studying AI due to several converging factors that promote chronic inflammation including high infection rate, elevated proinflammatory cytokine levels, uremia and arteriosclerosis^10^. The uremic milieu itself generates persistent immune activation through accumulated uremic toxins, oxidative stress, and metabolic acidosis^11^. Moreover, the HD procedure *per se* further exacerbates inflammation through vascular access, biocompatibility reactions with dialysis membranes, and endotoxin exposure from dialysate^4,12^.

This sustained inflammatory state, combined with ongoing blood losses during dialysis and potential iron deficiency, together with dysregulation of iron metabolism, contributes to AI pathogenesis, which is characterized by increased levels of ferritin, reduced iron and total iron-binding capacity (TIBC), together with high levels of iron in the bone marrow^13^. Noteworthy, anemia occurs in the early stages of CKD, and its incidence increases as renal function declines^14^. The etiology of anemia in CKD patients is multifaceted. The main causative factors are functional or absolute iron insufficiency, relative erythropoietin (EPO) deficiency, low levels of vitamin B complex and folic acid, chronic inflammation, infection, and iatrogenic blood loss^15^, in addition to the reduced lifespan caused by increased erythrophagocytosis by cytokine-activated macrophages^16^.

The pathogenesis of AI involves a complex interplay between regulators of iron metabolism and inflammatory mediators that ultimately disrupts erythropoiesis. Pro-inflammatory cytokines like interleukin6 (IL-6) plays central roles in this process by inducing hepatic production of hepcidin, the master regulator of iron homeostasis^17^. Hepcidin targets iron exporter ferroprotein and therefore, limits the entry of elementary iron into the systemic circulation. It is produced primarily in the liver cells in response to increased iron reserves or inflammatory signals. Iron induces the synthesis of bone morphogenetic protein 6 (BMP6) in liver sinusoidal endothelial cells, that binds to BMP receptors in hepatocytes and induces SMAD signal cascade, which in turns stimulates the transcription of the hepcidin antimicrobial peptide (HAMP) gene that encodes hepcidin. The signaling of SMAD is also crucial for inflammatory induction of HAMP gene through the IL-6/Signal transducer and activator of transcription 3 (STAT3) pathway^18^. In addition, CKD associated inflammation directly exacerbates anemia via inhibiting the production of EPO^19^.

Current evidence designates the important role of microRNAs (miRNAs) in inflammatory responses and many aspects of iron homeostasis. MiRNAs represent a class single-stranded non-coding RNAs that usually contain a small number of nucleotides (19–24 nucleotides in length). They control target gene expression, especially at the translational level. During inflammation, the expression patterns of miRNAs is dysregulated, resulting in the development of hyper-inflammatory phenotype. Inflammatory mediators, cytokines, chemokines and various proteins can regulate the expression of miRNAs, potentially contributing to the regulation of multiple genes and gene regulatory networks associated with inflammation. Noteworthy, miRNAs are crucial for the development, differentiation, function and survival of various immune cells including B and T lymphocytes, dendritic cells and macrophages^20^. Despite the growing recognition of miRNAs as important regulators of inflammation and iron metabolism including miR-34, miR-130 and miR-16b, their specific roles in HD-associated AI remain poorly characterized. Therefore, unravelling the role of miRNAs in the coordination of molecular responses to changes in iron status provides fundamental insights into how iron homeostasis is maintained^20^.

The National Institute of Health and Care Excellence (NICE) recommends using iron or erythropoiesis stimulants (ESAs), or both in combination, to treat CKD associated anemia. This aims to address both the absolute and functional deficiency of iron^17^. Furthermore, because ESA therapy increases erythropoiesis, this results in iron depletion, which causes a relative deficiency of iron. It’s imperative to develop new diagnostic and predictive biomarkers that enable personalized and accurate therapeutic management of AI in HD patients^21^. Anti-IL-6 drugs may be very effective in the management of AI in HD patients, reflecting the important role of IL-6 in the disease pathogenesis^22^. Unraveling the pathogenesis of AI has contributed to the continuous development of targeted therapies. For instance, Roxadustat (ROX) is a novel oral drug that has been recently approved for the treatment of anemia in HD patients. ROX inhibits the enzyme hypoxia-induced factor prolyl hydroxylase (HIF-PH) that inhibits hypoxia inducible factor (HIF) activity, an important transcription factor which controls tissue levels of oxygen. This in turns leads to an increase in the level of HIF, ultimately increasing the production of EPO^23^. Nevertheless, further elucidation of the underlying molecular mechanisms of AI could open new avenues for more effective management approaches than the current treatment modalities.

The interplay between cytokines and miRNAs in AI has not been extensively elucidated. The current study sought to investigate the intricate relationship between the expression of miR-34, miR-130 and miR-16b, and regulator genes of macrophage activity namely, IL-6 and TNF-α in HD patients suffering from AI, and evaluate their potential as promising biomarkers in the pathogenesis and progression of AI. Correlation analyses and pathway enrichment studies were performed to identify cytokine-miRNA regulatory networks that could unravel potential therapeutic targets to improve the management of this prevalent condition.

## Subjects and methods

### Biostatistical study design

Based on a previous study, the standard deviation (SD) of the control is 0.4 and the SD of the regression errors is 1.6. If the true slope of the line obtained by regressing patient’s against control is 1.3, 30 subjects for each group were needed to be able to reject the null hypothesis that this slope equals zero with probability (power) 95%. The Type I error probability associated with this test of this null hypothesis was 0.05.

### Ethics statement

The study protocol (PT611/28) was approved by the Ethics Committee of Theodor Bilharz Research Institute (TBRI), under the Federal Wide Assurance No. FWA00010609. All study participants read and signed written informed consents with the approval to use their blood specimens for research purposes. The study was conducted in compliance with the guidelines of the Declaration of Helsinki^24^. Confidentiality and personal privacy were respected in all levels of the study, and patients were informed to feel free to withdraw any time from the study without any consequences.

### Study design

Case-controlled, cross-sectional, observational single-center study.

### Study participants recruitment

The study population comprised 30 with established guideline conceptsadult HD patients recruited from the Hemodialysis Unit, Nephrology Department, TBRI, during the study period from 1^st^ January 2023 to 21^st^ March 2024. Patients were screened consecutively among adults attending regular maintenance HD and were enrolled only if they fulfilled the predefined inclusion criteria for AI, defined operationally based on a combination of hematologic, iron profile, and inflammatory markers consistent with functional iron deficiency, in accordance with established guideline concepts of KDIGO 2021^25^, and none of the exclusion criteria. Inclusion criteria were: (a) Age > 18 years, (b) On HD > 3 months, (c) AI defined as hemoglobin (Hb) < 10 g/dL, with Transferrin saturation (TSAT) < 20%, and ferritin > 300 ng/mL, and (d) Elevated CRP (> 5 mg/dL). Exclusion criteria included: (a) Active infection, (b) Iron/B12/folate deficiency, (c) Recent blood transfusion (< 3 months), (d) Severe primary cardiopulmonary failure, and (e) Malignancy.

Thirty healthy controls were recruited during the same period from volunteers with no history of kidney disease and not receiving any medications or suffering from any other diseases.

### Medication history

Patients were managed according to standard guideline-based care for anemia in maintenance HD, consistent with KDIGO 2021 Clinical Practice Guidelines^25^. This included the use of ESAs in patients with Hb levels below 10 g/dL, targeting a Hb range of 10–11.5 g/dL. I.V. iron supplementation was administered as clinically indicated to optimize iron availability, particularly in patients with low TSAT < 20%, despite elevated ferritin levels consistent with functional iron deficiency. Treatment decisions were individualized based on iron indices, inflammatory status, and clinical response.

### Samples collection

Venous blood samples were collected from 30 healthy subjects, and 30 HD patients from the HD unit, Nephrology Department, TBRI. Five milliliters of blood were aseptically withdrawn from each patient before the start of HD session, via venipuncture into 3 vacutainer tubes, two EDTA tubes (2 × 2 mL), one tube was used to perform complete blood count (CBC). The second EDTA tube was immediately used for molecular testing. One serum separator tube (1 × 1 mL) was collected for blood chemistry, iron profile and CRP. The serum tube was left to clot for 10 min, followed by centrifugation at 3000 rpm for 15 min and the serum was kept at −80℃ for further analysis.

### Laboratory investigations

CBC and differential white blood cells (WBCs) count were performed on DXH 500, Beckman Coulter, USA. Serum creatinine, urea, iron profile (serum iron, TIBC and ferritin), and CRP, were all performed on automated chemistry analyzer Cobas C 311, Roche, Switzerland.

### Bioinformatics study design

The study design was based on the integrated online databases for annotated and predicted human genes GeneCards https://www.genecards.org. The knowledgebase automatically integrates gene-centric data from ~ 150 web sources, including genomic, transcriptomic, proteomic, genetic, clinical and functional information. Among the macrophage activity as a selection criterion, the selected genes were “TNF, IL-10, IL-6, IFNG, PPARG, AKT1, TLR4, and TGFB1”, while the output depending on anemia in chronic renal failure cases were “TNF-α and IL-6”. Interestingly, the output depending on the gene regulators to the macrophage activity were “TNF-α and IL-6”.

**Fig. 1:**
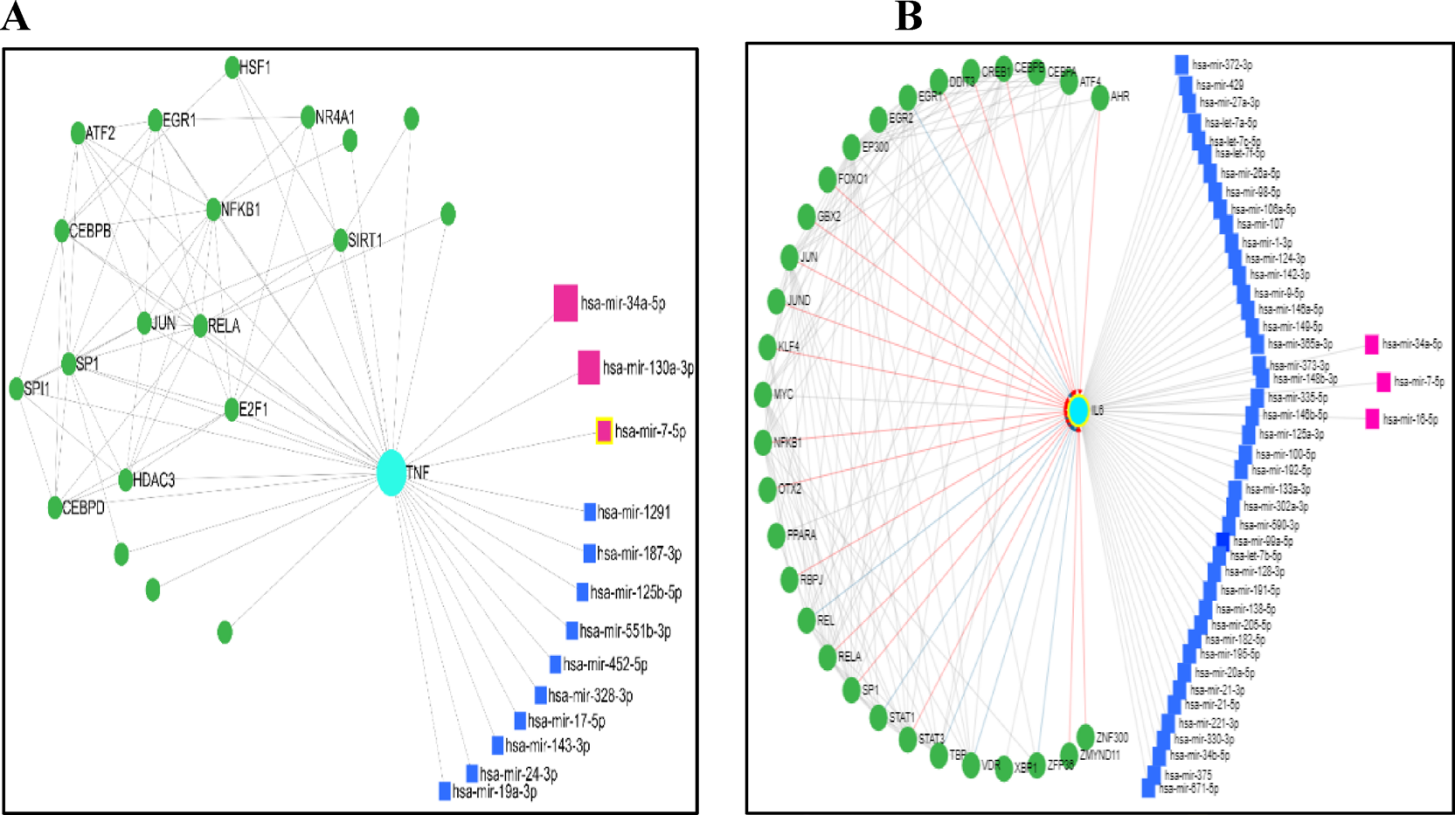
Outlook of all possible functional target miRNAs to (**A**) TNF-α gene and (**B**) IL-6 gene by miRNet webserver database among anemic chronic renal failure cases.

According to previous studies, miR-34, miR-320, miR-122, miR-130, miR-451, miR-23a, and miR-16b were shown to be related to the regulation of anemia in chronic renal failure cases. After the bioinformatics analysis was performed using miRNet version 2.0 *“*https://www.mirnet.ca”, which act as a miRNA-centric network visual analytics platform, we found that miR-34, miR-130 and miR-7 were the most significant miRNAs related to the TNF-α gene in anemic chronic renal failure cases (Fig. 1A), while miR-34, miR-16 and miR-7 were the most significant miRNAs related to the IL-6 gene in anemic chronic renal failure cases (Fig. 1B).

Accordingly, we aimed to study the relations of miR-34, miR-130, miR-16b and gene expression of TNF-α and IL-6 genes, which act as the regulator genes to the macrophage activity in chronic renal failure cases with anemia, in addition to studying the interactions between them.

To explore the potential regulatory role of hsa-miR-16, hsa-miR-34, and hsa-miR-130 in inflammatory cytokine expression, we utilized publicly available datasets via https://www.cbioportal.org/ (Accessed on April 9th, 2025)^26^. Specifically, we investigated associations between the expression levels of our investigated miRNAs and the two pro-inflammatory cytokines, IL-6 and TNF-α, within the TCGA KIRC dataset, which provides both miRNA and mRNA sequencing data. Correlation analyses were performed using the LinkedOmics integration, which applies Pearson correlation to identify statistically significant relationships. Data were log2-transformed, and results were adjusted for multiple testing using the Benjamini-Hochberg (BH) method to calculate the False Discovery Rate (FDR).

### Molecular gene expression of cytokines and microRNAs

#### RNA extraction

The miRNeasy extraction kit was used to extract total RNA (Qiagen, Valencia, CA) from serum samples, in accordance with the manufacturer’s instructions. Samples were extracted in duplicates and quantitated using a Nanodrop ND-2000c (Thermo Scientific, Waltham, MA, USA), by measuring absorbance at 260/280 nm. The extracted RNA were stored at − 80 °C for further analyses.

#### MicroRNA extraction

The mirVana Kit^®^ (Applied Biosystems, CA, USA) was utilized to isolate miR-34, miR-130, and miR-16b from plasma samples, following the instructions of the manufacturer. Samples were also quantified spectrophotometrically.

#### Real-time quantitative reverse-transcription polymerase chain reaction  (qRT-PCR) assay

The extracted RNA samples (5 µl), were reverse transcribed using the QuantiTect Reverse Transcription Kit (Qiagen, Valencia, CA). The real time PCR amplification step was carried out using specific primers (Qiagen, Hilden, Germany) using 5 µl of the cDNA and using the normalizing control U6. The PCR program was set as follows: 10 min at 25 °C, 120 min at 37 °C, and 5 min at 85 °C, using the thermal cycler (BioRad-T 100, Singapore).

The expression of IL-6 and TNF-α genes was measured by qRT-PCR assay using (Maxima SYBR Green/ROX qPCR Master Mix 2X, Thermo Fisher, UK) in Applied Biosystems™ StepOne™ Real-Time PCR System. GAPDH was used as a normalizing control. The PCR program cycle comprised initiation for 10 min at 95 °C, followed by 15 s at 95 °C, then 30 s at 55 °C, and 15 s at 72 °C, and the last steps were repeated 45 times. Samples were run in duplicates, and the ΔΔCT method was used for the relative quantification of miRNAs, TNF-α and IL-6 in all samples^27^. The sequences of the employed primers are depicted in Table 1.

**Table 1:** Sequence of primers used for qRT-PCR.

Gene	Forward primer (5ʹ–3ʹ)	Reverse (3ʹ–5ʹ)
miR-34	GGACGGTAGCAAGCAAAGAGTGTGACAACCAGCTA	GGGATTCTGGAAGATGATGATGACTGGCAGTGTCT
miR-130	GGACGGTAGCAAGCAAAGAGTGTGAGTAGCACAAT	GGGATTCTGGAAGATGATGATGACGCTCTTTTCAC
miR-16b	GGACGGTAGCAAGCAAAGAGTGTGCGCCAATATTT	GGGATTCTGGAAGATGATGATGACTAGCAGCACGT
TNF-α	TCTCATGCACCACCATCAAGGACT	ACCACTCTCCCTTTGCAGAACTCA
IL-6	ATCCAGTTGCCTTCTTGGGACTGA	TAAGCCTCCGACTTGTGAAGTGGT
U6	CTCGCTTCGGCAGCACA	AACGCTTCACGAATT TGCGT
GAPDH	ATTCCATGGCACCGTCAAGGCTGA	TTCTCCATGGTGGTGAAGACGCCA

### Statistical analysis

The data were analyzed using Microsoft Excel 365 and a statistical package for social science, ‘IBM SPSS Statistics, version 29.0.1.1 (IBM Corp., Armonk, N.Y., USA)’. Continuously normally distributed variables were represented as mean ± SD, with a 95% confidence interval, and based on the frequencies and percentages for categorical variables, a *p*-value < 0.05 was considered statistically significant. A student’s *t*-test was used to compare the means of normally distributed variables between groups, while the chi-squared (X^2^) test was performed to compare groups in categorical variables. The diagnostic performance of the studied markers was assessed by receiver operating characteristic (ROC) curves. The area under the ROC curve (AUC) was calculated as an accuracy index for the prognostic performance of selected tests. Effect modifications were evaluated by stratification, and statistical interaction was assessed by including the main effect variables and their product terms in the logistic regression model. Pearson correlation analysis was performed to assess the strength of the association between the two markers.

## Results

### Demographic and clinical characteristics of study participants

This study involved 60 participants, including 30 HD patients with AI, and 30 healthy volunteers with no history of kidney disease serving as a control group. The clinical data of all participants are shown in Table 2. The HD with AI group subjects had mean age (53.7 ± 10.9 years), including 21 (70%) males and 9 (30%) females, while the healthy volunteers group subjects were with mean age (53.2 ± 8.8 years), including 15 (50%) males and 15 (50%) females. Demographic and clinical characteristics of the enrolled subjects are displayed in Table 2.

**Table 2:** Demographic and clinical characteristics of patients and healthy volunteers.

Demographic and Clinical data (Unit)		Control (N=30)	HD (N=30)	*p*-value
Age (Years)		53.2±8.8	53.7±10.9	0.846
Gender	Female	15(50.0%)	9(30.0%)	0.094
	Male	15(50.0%)	21(70.0%)	
Cause of ESRD	Acute parenchymal kidney disease	-	3(10.0%)	-
	Hypertension	-	16(53.3%)	-
	Nephrocalcinosis	-	3(10.0%)	-
	NSAID abuse	-	3(10.0%)	-
	Obstructive	-	3(10.0%)	-
	Tubulointersitial Nephritis	-	2(6.7%)	-
HD Duration (Years)		-	8.4±5.2	-
Liver Ultrasound	Normal	-	26(86.7%)	-
	Fatty	-	4(13.3%)	-
Liver (cm)		-	13.7±1.0	-
Spleen size (cm)		-	11.4±1.3	-
AST (U/L)		20.3±5.1	20.5±7.0	0.916
ALT (U/L)		18.7±9.8	17.0±4.5	0.391
Serum albumin (g/dL)		4.4±0.2	4.1±0.3	<0.001**
Creatinine (mg/dL)		0.8±0.2	8.4±2.1	<0.001**
Urea (mg/dL)		21.0±5.3	128.7±25.0	<0.001**
Hb (g/dL)		14.2±1.7	8.7±1.1	<0.001**
Hct		43.6±4.7	27.9±3.9	<0.001**
RBCs		5.2±0.5	3.5±0.6	<0.001**
MCV		82.2±5.1	77.1±5.7	<0.001**
MCH		27.1±2.2	25.6±3.2	0.032*
MCHC		33.0±1.6	32.3±1.3	0.052
RDW		13.7±0.8	17.0±3.0	<0.001**
TLC		7.7±1.2	6.9±1.8	0.067
Neutrophils		58.0±8.6	63.2±8.6	0.023*
Lymphocytes		37.0±8.5	24.7±5.2	<0.001**
Monocytes		3.3±1.0	8.1±4.3	<0.001**
Eosinophils		1.3±0.5	3.7±3.7	<0.001**
Basophils		0.3±0.5	0.6±0.5	0.095
Platelets		258.2±39.9	185.0±38.3	<0.001**
CRP (mg/L)		0.4±0.2	38.2±24.6	<0.001**
Ferritin (ng/mL)		72.3±43.0	547.8±138.4	<0.001**
Trans%		36.8±6.8	13.1±5.6	<0.001**
TIBC (μg/dL)		333.7±41.8	313.8±63.5	0.159
Iron (μg/dL)		124.2±33.3	46.7±26.3	<0.001**

ALT, Alanine transaminase; AST, Aspartate transaminase; CRP, C-reactive protein; ESRD, End-stage renal disease; HD, Hemodialysis; Hb, Hemoglobin; Hct, Hematocrit; MCV, Mean corpuscular volume; MCH, Mean corpuscular hemoglobin; MCHC, Mean corpuscular hemoglobin concentration; NSAID, Non-steroidal anti-inflammatory drug; RBCs, Red blood cells; RDW, Red cell distribution width; TIBC, Total iron binding capacity; TLC, Total leukocyte count; Trans%, Transferrin saturation. Age, HD Duration, Hb, Hct, RBCs, MCV, MCH, MCHC, RDW, TLC, Neutrophil, Lymphocyte, Monocyte, Eosinophils, Basophils, Platelets, CRP, Ferritin, Trans%, TIBC, and Iron are represented as Mean ± SD; the data were analyzed by t test. Gender, cause of ESRD, and Liver Ultrasound are represented as Frequency and percentage F(%); the data were analyzed by X^2^ test.

* *p *value < 0.05 is significant, ** *p *value < 0.01 is highly significant.

### Expression of IL-6, TNF- α, miR-34, miR-130, miR-16b in peripheral blood of HD patients with AI and controls

**Fig. 2:**
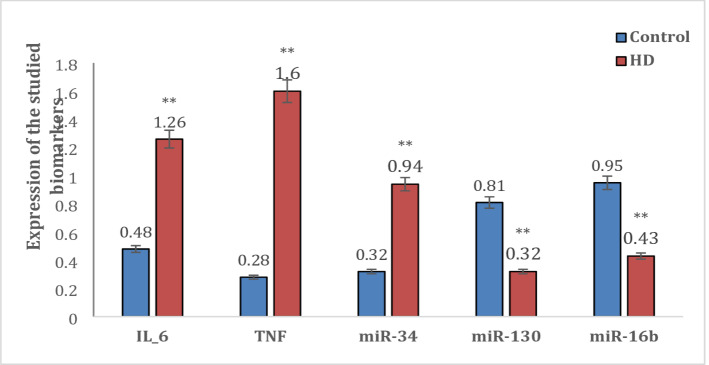
Expression of the studied biomarkers in the HD with AI and control groups.

The expression of IL-6, TNF-α, miR-34, miR-130, miR-16b in peripheral blood of HD patients with AI and controls is represented as mean ± SD. IL-6, TNF-α and miR-34 showed significant overexpression (*p* < 0.001) in HD with AI compared to control (1.26 ± 0.08 vs. 0.48 ± 0.03, 1.6 ± 0.07 vs. 0.28 ± 0.04 & 0.94 ± 0.05 vs. 0.32 ± 0.07, respectively). On the other hand, miR-130 and and miR-16b showed significant lower expression (*p* < 0.001) in HD with AI compared to control (0.32 ± 0.05 vs. 0.81 ± 0.04 & 0.43 ± 0.04 vs. 0.95 ± 0.03, respectively) (Fig. 2).

### Correlation analysis of IL-6, TNF- α, miR-34, miR-130 and miR-16b expression

Pearson correlation analysis was performed within the HD patients with AI group (*n* = 30) to assess the relationship between IL-6, TNF-α, miR-34, miR-130, and miR-16b expression levels. A positive significant correlation was observed between IL-6 and miR-34 (*r* = 0.963, *p* < 0.001), while it showed significant inverse correlations with miR-130 (*r*= −0.979, *p* < 0.001) and miR-16b (*r*=−0.981, *p* < 0.001). Regarding TNF-α, a positive significant correlation was observed with miR-34 (*r* = 0.981, *p* < 0.001), and inverse significant correlations were noted with both miR-130 and miR-16b (*r*=−0.976, −0.992 respectively, *p* < 0.001 for both). Inverse correlations were observed between miR-34 and both miR-130 and miR-16b (*r*=−0.972 and − 0.967 respectively, *p* < 0.001). Regarding the Pearson correlation analysis between miR-130 and the other 2 studied miRNAs, an inverse significant correlation was noted with miR-34 (*r*=−0.972), while a significant positive one was observed with miR-16b (*r* = 0.973, *p* < 0.001). Finally, for miR-16b, an inverse significant correlation was observed with miR-34 (*r*=−0.976), while a significant positive one was observed with miR-130 (*r* = 0.973,  *p* < 0.001for both) (Supplementary Table 1).

### The association between the expression of IL-6, TNF- α, miR-34, miR-130, and miR-16b regarding the degree of anemia in HD with AI

The results of the study indicated no association between degree of anemia and the expression of IL-6, TNF-α, miR-34, and miR-130 genes. On the other hand, there was a positive direct association between marked anemia (< 7 g/dl) and the decreased expression of miR-16b (*p* = 0.048). Furthermore, direct positive association was shown between lower levels of TSAT% (< 10%) with decreased expression of TNF-α, and miR-130 (*p* < 0.001 for both). In contrast, miR-16b increased in the reverse direction (*p* = 0.002). The markedly increased ferritin levels (> 600ng/ml) showed direct positive association with increased expression of IL-6, TNF-α and miR-130 (*p* < 0.001, 0.009, & <0.001, respectively). In contrast, mir-34 and miR-16b expression increased in the reverse direction (*p* < 0.001 and <0.001, respectively). In an interesting finding, higher levels of CRP (> 50 mg/dl) showed positive association with decreased expression of IL-6, TNF-α, and miR-16b (*p* < 0.001, 0.003, and <0.001, respectively). In contrast, miR-34 and miR-130 expression increased in the reverse direction (*p* = 0.013 and <0.001, respectively) (Table 3).

**Table 3:** The association between the expression of IL-6, TNF**-**α, miR-34, miR-130, and miR-16b in HD with AI categories.

Hb g/dl	< 10 − 7	< 7	*p* value
IL-6	1.25 ± 0.08	1.28 ± 0.08	0.23
TNF-α	1.60 ± 0.08	1.59 ± 0.05	0.28
miR-34	0.94 ± 0.05	0.92 ± 0.05	0.178
miR-130	0.32 ± 0.05	0.34 ± 0.05	0.054
miR-16b	0.43 ± 0.04	0.41 ± 0.04	0.048*
TSAT%	**< 20 − 10%**	**< 10%**	***p*** **value**
IL-6	1.26 ± 0.07	1.24 ± 0.10	0.33
TNF-α	1.63 ± 0.06	1.52 ± 0.01	< 0.001**
miR-34	0.94 ± 0.06	0.93 ± 0.04	0.268
miR-130	0.34 ± 0.04	0.26 ± 0.03	< 0.001**
miR16b	0.42 ± 0.04	0.45 ± 0.00	0.002**
**Ferritin ng/ml**	**300–600**	**> 600**	***p*** **value**
IL-6	1.24 ± 0.08	1.34 ± 0.00	< 0.001**
TNF- α	1.59 ± 0.07	1.64 ± 0.00	0.009**
miR-34	0.95 ± 0.05	0.87 ± 0.00	< 0.001**
miR-130	0.31 ± 0.05	0.37 ± 0.00	< 0.001**
miR-16b	0.43 ± 0.04	0.40 ± 0.00	< 0.001**
**CRP mg/dl**	**6–50**	**> 50**	***p*** **value**
IL-6	1.27 ± 0.08	1.17 ± 0.00	< 0.001**
TNF-α	1.60 ± 0.07	1.55 ± 0.00	0.003**
miR-34	0.93 ± 0.05	0.96 ± 0.00	0.013*
miR-130	0.31 ± 0.05	0.37 ± 0.00	< 0.001**
miR-16b	0.43 ± 0.03	0.38 ± 0.00	< 0.001**

The expression of the studied biomarkers is represented as mean ± SD; the data were analyzed by *t* test. * *p* value < 0.05 is significant, ** *p* value < 0.01 is highly significant.

### Diagnostic significance of IL-6, TNF-α, miR-34, miR-130, and miR-16b in HD with AI

The ROC curve analysis denoted the promising diagnostic value of miR-34, miR-130 and miR-16b in HD with AI with cut off values of (0.64, 0.57 & 0.62, respectively). AUC was 100, (95% CI), (lower bound-upper bound = 100–100), 100% sensitivity and 100% specificity for all. This indicates that those biomarkers have the potential to be used as new noninvasive diagnostic biomarkers for HD patients with AI (Supplementary Fig. 1).

### Prognostic significance of miR-34, miR-130, and miR-16b in HD with AI

After adjustment for patients’ age and gender using logistic regression analysis (Supplementary Table 2), there was a statistically significant relation between anemia and higher expression of miR-34 (OR = 3.143, 95% CI = 0.896–6.547, *p <* 0.001). On the other hand, there was statistically significant relation between anemia and lower expression of miR-130 (OR = 0.144, 95% CI = 0.025–0.867, *p <* 0.001). A similar trend was demonstrated for low miR-16b expression (OR = 0.125, 95% CI = 0.048–0.769, *p <* 0.001).

### *In silico* bioinformatics analysis

**Fig. 3:**
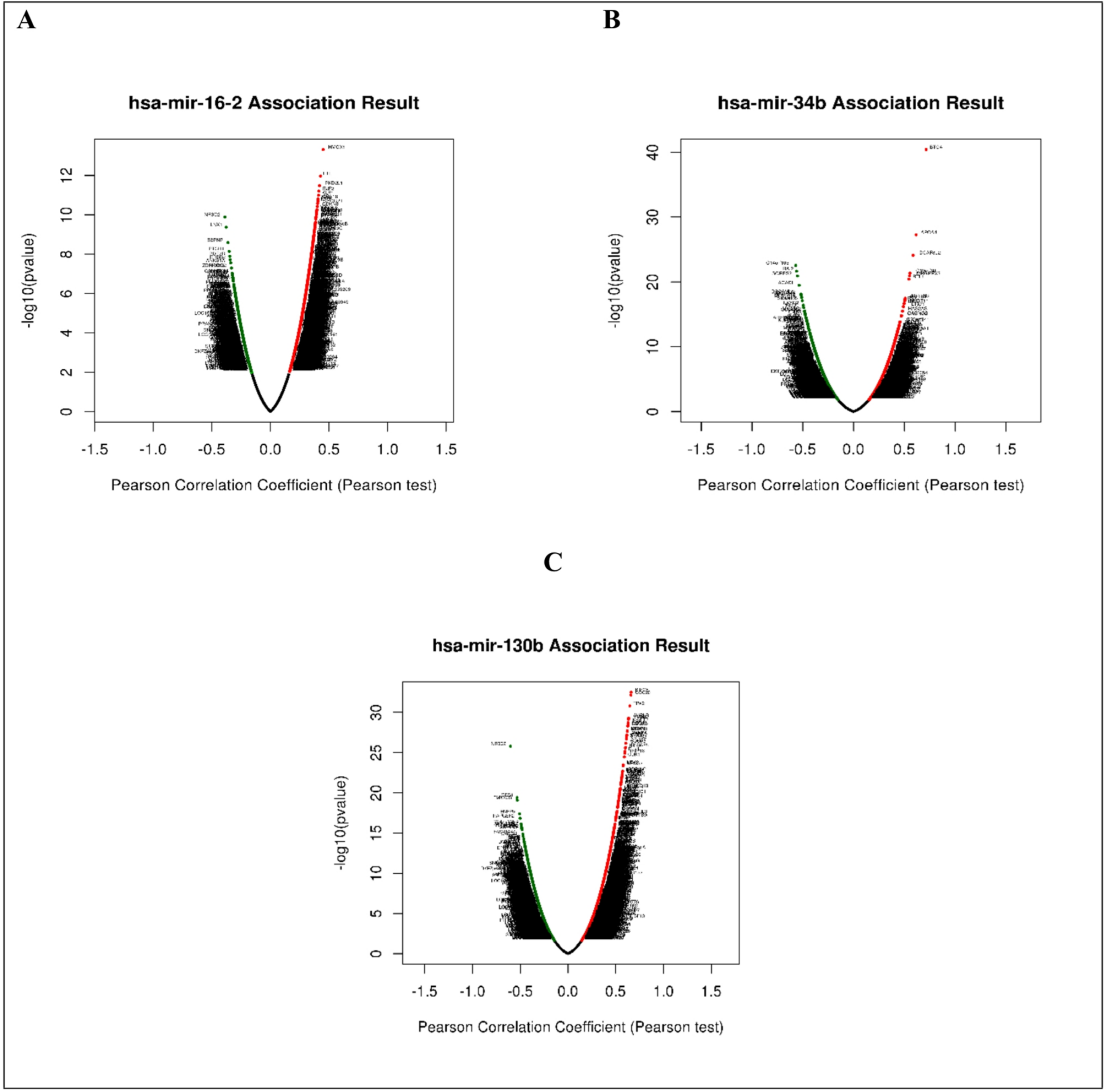
Pearson correlation plots between each of the investigated miRNAs (miR-16, miR-34, miR-130) and all expressed genes in the TCGA KIRC dataset.

**Fig. 4:**
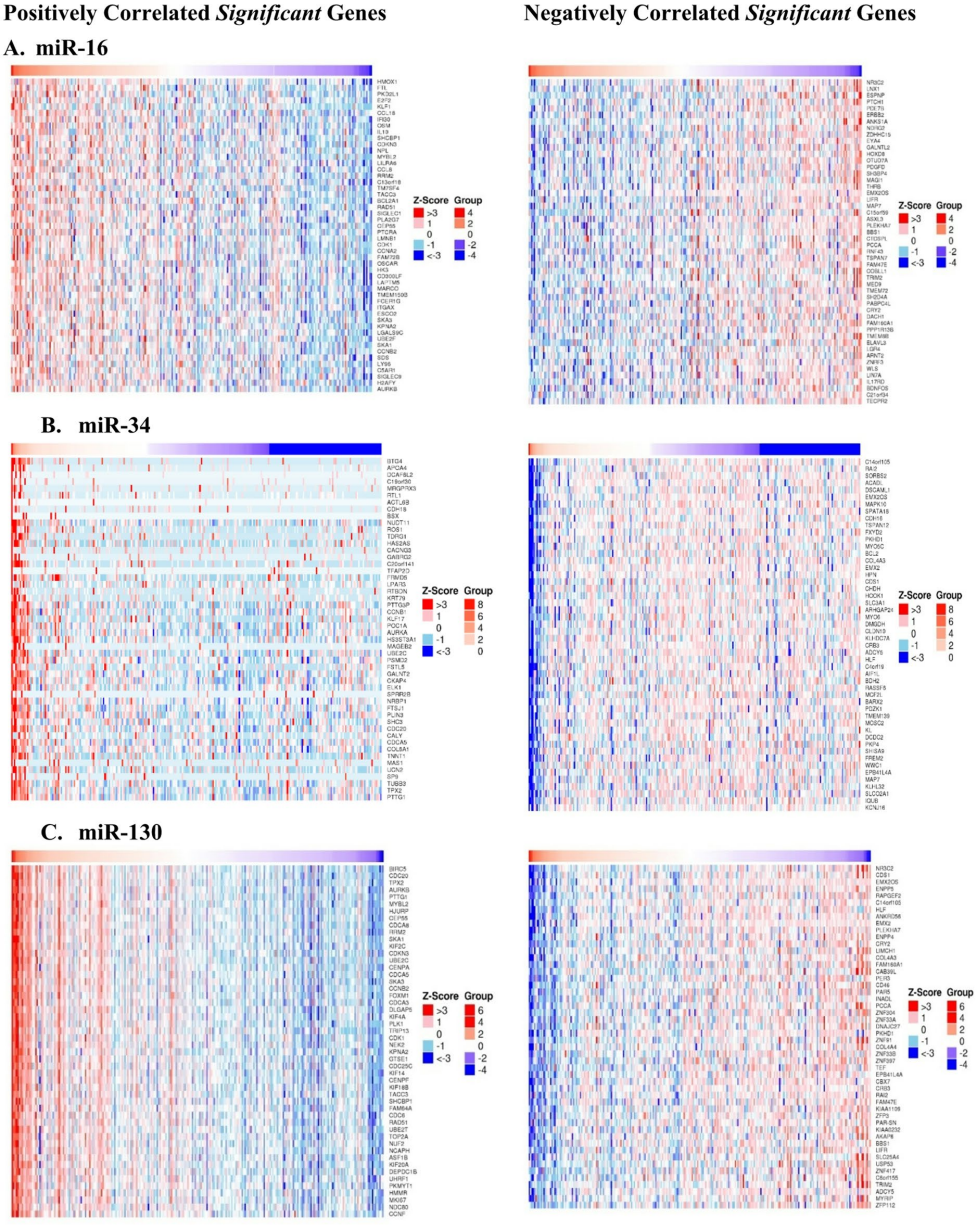
Heatmaps showing positively and negatively correlated significant genes for each miRNA. (**A**) miR-16, (**B**) miR-34, (**C**) miR-130.

Using cBioPortal (https://www.cbioportal.org/), we were able to identify the correlation between our investigated miRNAs, miR-16b, miR-34 and miR-130 and all expressed genes related to renal failure. Figure 3 shows the Pearson correlation plot between each of the studied miRNAs and more than 102.5 M genes associated with TCGA KIRC dataset. A heat plot for the positively and negatively correlated *significant* genes against each miRNA are shown in Fig. 4.

**Fig. 5:**
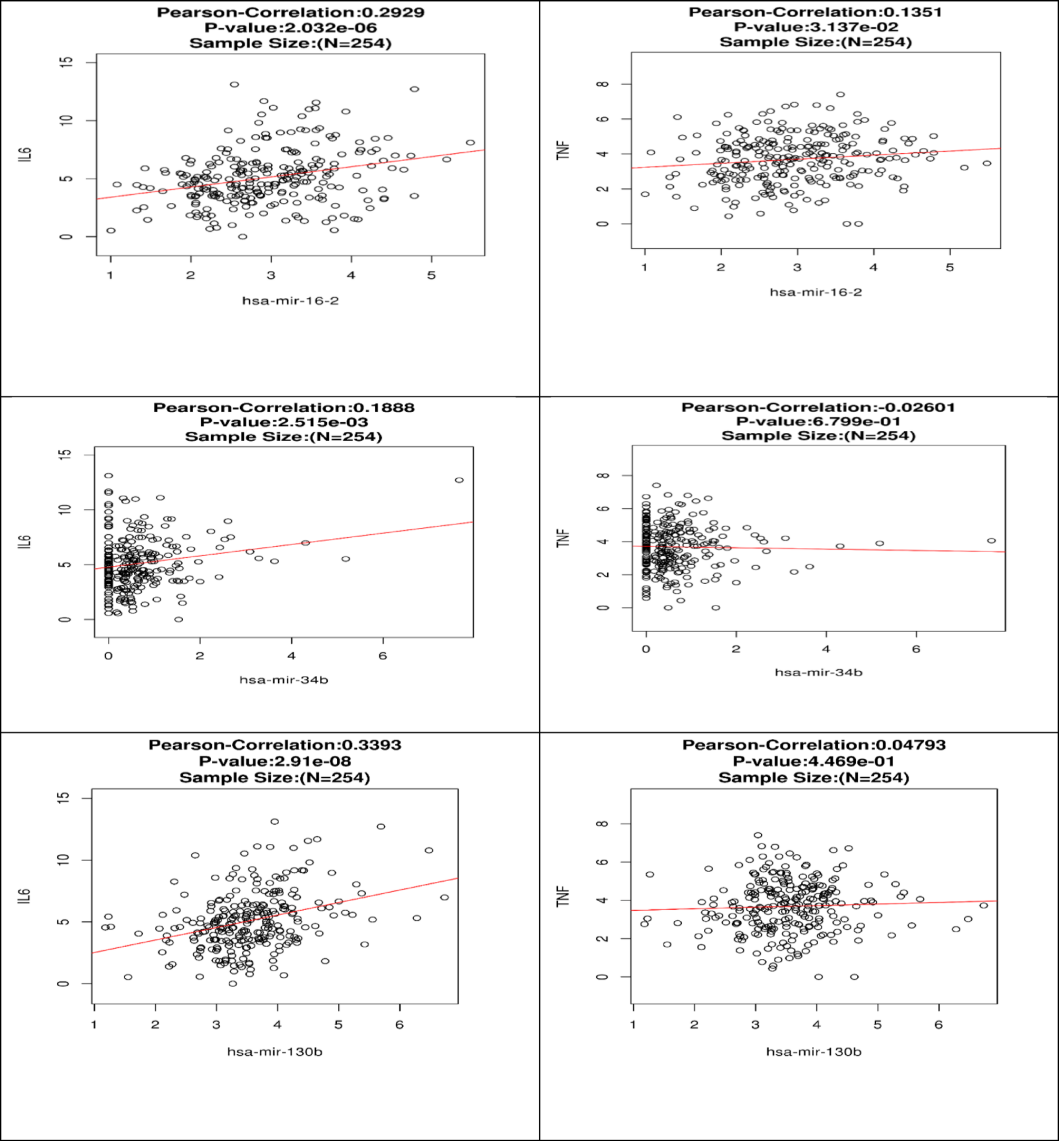
Scatter plots showing the Pearson correlation between expression levels of each investigated miRNA and the pro-inflammatory cytokines IL-6 and TNF-α.

The Pearson correlation between the expression levels of each of our investigated miRNAs and the two pro-inflammatory cytokines, IL-6 and TNF-α were extracted and listed in Supplementary Table 3 and Fig. 5.

Correlation analyses revealed a moderate positive association between hsa-miR-16 and IL-6 expression (Pearson correlation coefficient = 0.293). This correlation was statistically significant with a *p*-value of 2.03 × 10⁻⁶ and remained significant after FDR correction (FDR = 9.38 × 10⁻⁵), based on 254 and 253 valid samples for hsa-miR-16 and IL-6, respectively.

Conversely, the correlation between hsa-miR-16 and TNF-α was weaker (*r* = 0.135). While the initial *p*-value (0.031) suggested marginal statistical significance, this did not hold after FDR adjustment (FDR = 0.118), reducing confidence in the robustness of this association. A total of 254 and 252 samples were analysed for hsa-miR-16 and TNF-α, respectively.

Our results also showed a weak but statistically significant positive correlation between miR-34b and IL-6 expression (*r* = 0.189), with a *p*-value of 0.0025 and an FDR (Benjamini-Hochberg) of 0.013, across 177 and 253 valid samples for miR-34b and IL-6, respectively.

In contrast, the correlation between miR-34b and TNF-α was statistically non-significant (*r* = −0.026, *p* = 0.680, FDR = 0.800) based on 177 and 252 valid samples for miR-34b and TNF-α, respectively.

And finally, a moderate positive correlation between hsa-miR-130b and IL-6 expression (*r* = 0.339) was detected, which was highly statistically significant (*p* = 2.91 × 10⁻⁸) and remained significant after multiple testing correction (FDR = 5.52 × 10⁻⁷). The analysis was conducted across 254 samples for miR-130b and 253 for IL-6. In contrast, the association between miR-130b and TNF-α was very weak (*r* = 0.048) and not statistically significant (*p* = 0.447, FDR = 0.561), based on 254 and 252 valid samples, respectively.

## Discussion

The exact mechanisms for the development of AI in HD can be linked to dysregulated iron homeostasis that leads to a reduction in iron absorption and its release from the RES and, finally, hypoferremia^28^. IL-6 and TNF-α levels were reported to be increased in 30 to 50 % of CKD patients^13^. IL-6 increases the serum level of hepcidin, which blocks the binding of iron to the iron transporter ferroportin^29^.

To our knowledge, this is the first study to explore the interplay between the expression of cytokine genes and miRNAs in the context of AI in HD patients. The literature about the role of miRNAs in HD with AI is scarce. However, other research groups investigated these biomarkers in other types of anemia and diverse inflammatory disorders. The complex interplay between miRNAs and cytokines in the context of inflammation underscores the intricacy of AI in HD patients. MiRNAs dysregulation may exert both direct effects on iron metabolism and erythropoiesis, as well as indirect effects through the modulation of the inflammatory response.

The results of the present study showed significant overexpression of the cytokine genes, IL-6 and TNF-α, differentially accompanied by overexpression of miR-34 and significant lower expression of miR-130 and miR-16b in HD patients with AI compared to control. Moreover, the set of the studied miRNAs showed differential significant correlation with both cytokine genes. These results are aligned with a previous report from Lu *et al.*^30^, who identified the role of miR-34a-5p as a direct target for p53 transcription, which in turns stimulates T cell activation via the Wingless-related integration site (WNT), RAS-ERK, and nuclear factor kappa B (NF-κB) signaling pathways in aplastic anemia. Furthermore, Das and Rao^31^ reported that miR-34 induced the release of proinflammatory cytokines and chemokines by targeting leucine rich repeat containing G protein-coupled receptor 4 (LGR4) and its inhibition by upregulating WNT1 and Jagged Canonical Notch Ligand 1 (JAG1) to delay the differentiation of monocytes to dendritic cells.

On the other hand, Fouad *et al*.^32^, showed that the expression of miR-34a was significantly downregulated in inflammatory bowel disease patients compared to healthy controls. However, a significant positive correlation was observed between the expression of miR-34a and the levels of Hb. Similarly, Zhao *et al.*^33^ showed that dynamic expression of miR-34a was crucial to limiting reactive inflammation. Additionally, miR-34a deficiency increased the inflammation induced by IL-6R/STAT3. *In silico* analysis showed a statistically significant but weak positive correlation between miR-34b and IL-6 expression. While the effect size is limited, the significance after multiple testing correction suggests a potential regulatory or co-expressive relationship in modulating the inflammatory microenvironment, potentially through feedback regulation in the IL-6/STAT3 axis. Taken together, these findings may point toward a modest role of miR-34b in modulating IL-6-driven inflammatory pathways, which are especially relevant in inflammatory anaemias and renal pathologies.

The study by Zumbrennen-Bullough *et al.*^34^, found that miR-130a in mice liver was upregulated by low iron, and targets ALK2 to downregulate BMP-SMAD signals and the expression of hepcidin in liver cells. A positive correlation between miR-130b and IL-6 was observed via *in silico* analysis, suggesting a potential regulatory or co-expression relationship. Given the central role of IL-6 in inflammatory pathways, particularly in the AI observed in CKD and HD patients, this result implies that miR-130b could be involved in modulating IL-6 expression or participates in shared signalling cascades, such as the JAK/STAT or NF-κB pathways. Previous studies have implicated miR-130b in various inflammatory and oncogenic processes, including macrophage activation, and cytokine regulation^35^. Its correlation with IL-6 aligns with this role and may point to a post-transcriptional regulatory mechanism influencing the cytokine milieu in chronic inflammation.

Regarding miR-16, the study by Du *et al.*^36^ showed increased expression of miR-16 in serum of newborns with sepsis, inhibiting the inflammatory pathways induced by lipopolysaccharides (LPS), suggesting that miR-16 was associated with immune regulation. The inflammation reaction by NF-KB and Mitogen-activated protein kinase (MAPK) signals may be regulated by miR-16 by direct mediation of transforming growth factor beta activated kinase 1 (MAP3K7) binding protein 3 (TAB3). Accordingly, miR-16 could exert a protective role against LPS-induced inflammation. Hence, miR-16 represents a new therapeutic target for preventing inflammation. In addition, the bioinformatics analysis suggests a statistically significant moderate correlation between miR-16 and IL-6, indicating that miR-16 may play a regulatory role in modulating IL-6 expression in conditions associated with inflammation. IL-6 is a key mediator of the acute phase response and contributes to impaired iron metabolism, reduced erythropoiesis, and increased hepcidin levels in chronic inflammatory states^37^. These insights align with the understanding that miR-16 is involved in controlling genes related to cell cycle, apoptosis, and inflammation, and support its potential role as a post-transcriptional regulator of IL-6 in inflammatory conditions, including CKD-related anaemia.

The results of the current study indicated a positive direct association between the reduced expression of miR-16b and marked anemia (Hb < 7 g/dl). Furthermore, there was a direct positive association between decreased expression of miR-130 and lower levels of TSAT% (< 10%). Remarkably, increased expression of miR-130 showed direct positive association with the markedly increased ferritin levels (> 600ng/ml). Finally, decreased expression of miR-16b showed a positive association with higher levels of CRP (> 50 mg/dl). These data suggest that the studied set of biomarkers are active players in the progression of AI in HD.

After adjustment for patients’ age and gender using logistic regression analysis, the current study showed a statistically significant relation between anemia and higher expression of miR-34. On the contrary, there was a statistically significant relation between anemia and lower expression of miR-130 and miR-16b. The current findings suggest the potential diagnostic and prognostic utility of the investigated biomarkers as potential tools for AI in HD.

The study by Dziedzic *et al.*^38^ supports these findings by reporting that miRNAs are easily accessible for analysis from body fluids. Consequently, circulating miRNAs could be potential noninvasive molecular biomarkers for different pathological conditions. Furthermore, miRNAs are relatively stable and resistant to degradation, making them ideal sources for large-scale clinical use. In addition, the detection of circulating miRNAs can provide additional information on specific pathological mechanisms in HD patients.

A previous study by Bowry and Gatti^39^ reported that the optimization of dialysis and integration with pharmaceutical interventions can overcome some of the current challenges in the control of anemia in HD patients, who are currently almost entirely dependent on externally expensive ESA/iron treatments. In addition, Theurl *et al.*^40^ reported that iron sequestration in the RES is the main pathophysiological inducer of AI. Therefore, prospectively investigating the effects of the iron mobilization using innovative drugs to reverse AI in HD will be of utmost importance using the studied miRNA network.

The altered expression of miRNAs in HD patients may contribute to the development of AI through the exacerbation of the inflammatory response, as well as via targeting key genes involved in iron metabolism and erythropoiesis which can directly impact RBCs production and function. The multifaceted nature of miRNA involvement suggests their potential utility as biomarkers for disease progression in AI. Further elucidation of specific miRNA signatures associated with this condition could facilitate the development of targeted interventions addressing both hematological and inflammatory aspects of the disease. In addition, miRNA mimics or inhibitors, may offer new avenues for managing anemia in this patient population. Such strategies could potentially address the underlying molecular mechanisms of AI more effectively than current treatments, which primarily focus on iron supplementation and erythropoiesis stimulation.

Some limitations must be considered when interpreting our findings. As a cross-sectional study, the results suggest possible relationships between miRNA dysregulation and the development or progression of AI, rather than causal association. They provide a basis for future longitudinal studies (with repeated sampling before and after clinical events like initiation of dialysis, ESA therapy, and iron supplementation) to delineate temporal relationships. Futher mechanistic studies are also warranted to clarify the underlying biological mechanisms and clinical relevance of these biomarkers. The single-center recruitment limits the generalizability to dialysis populations with different demographic characteristics, comorbidities, or dialysis modalities. The relatively small sample size, while adequate for primary analyses, limits statistical power for subgroup evaluations, and may limit the generalizability of the logistic regression findings; therefore, the present results should be interpreted as preliminary and are intended to provide a rationale for future large-scale, adequately powered prospective studies. In order to reduce the risk of selection bias, recruitment was based on consecutive screening where patients meeting eligibility criteria were approached consecutively during their scheduled dialysis sessions, with no selective sampling based on disease severity, inflammatory markers, or clinical characteristics was employed.

Our results should be interpreted as hypothesis-generating rather than affirmatory. While the observed miRNA expression patterns and logistic regression associations are biologically plausible and statistically significant within this cohort, they provide preliminary evidence that warrants confirmation through larger, multicenter, prospective validation studies to establish the basis for clinical adoption as biomarkers for AI in HD patients.

## Conclusions

The present study suggests an exploratory coordinated cytokine–miRNA regulatory network that may contribute to the pathophysiology of AI in HD patients. Increased expression of IL-6 and TNF-α was associated with differential expression of miR-34, miR-130, and miR-16b, reflecting a potential link between inflammatory signaling and disrupted iron homeostasis and erythropoiesis and suggest these molecules as candidates for further biomarker investigation and validation. Future interventional studies targeting this cytokine-miRNA axis may offer novel approaches for enhancing the efficacy of anemia management in HD patients.

## Supplementary Information

Below is the link to the electronic supplementary material.

Supplementary Material 1

## Data Availability

All data supporting the findings of this study are available within the paper.

## Abbreviations

AI: Anemia of inflammation
AUC: Area under the curve
BH: Benjamini-Hochberg
BMP6: Bone morphogenetic protein 6
CBC: Complete blood count
CKD: Chronic kidney disease
CRP: C- reactive protein
EPO: Erythropoietin
ERK: Extracellular signal-regulated kinases
ESAs: Erythropoiesis stimulants
ESRD: End stage renal disease
FDR: False Discovery Rate
HAMP: Hepcidin antimicrobial peptide
Hb: Hemoglobin
Hct: Hematocrit
HD: Hemodialysis
HIF-PH: Hypoxia-induced factor prolyl hydroxylase
HIF: Hypoxia inducible factor
IDA: Iron deficiency anemia
IL-6: Interleukin 6
JAG1: Jagged Canonical Notch Ligand 1
JAK1: Janus kinase 1
LGR4: Leucine-rich repeat-containing G-protein coupled receptor 4
LPS: Lipopolysaccharide
MCH: Mean corpuscular hemoglobin
MCV: Mean corpuscular volume
MCHC: Mean corpuscular hemoglobin concentration
MAPK: Mitogen-activated protein kinase
miRNAs: MicroRNAs
NF -kB: Nuclear factor kappa B
NICE: The National Institute of Health and Care Excellence
NSAID: Non-steroidal anti-inflammatory drug
PAN: Poly(A) nuclease
qRT-PCR: Quantitative Reverse Transcription Polymerase Chain Reaction
RBCs: Red blood cells
RDW: Red cell distribution width
RES: Reticuloendothelial system
ROC: Receiver operating characteristic curve
ROX: Roxadustat
SD: Standard deviation
STAT3: Signal transducer and activator of transcription 3
TAB3: Transforming growth factor-beta activated kinase 1 (MAP3K7) binding protein 3
TIBC: Total iron-binding capacity
TLC: Total leukocyte count
TNF-α: Tumor necrosis factor alpha
TSAT%: Transferrin saturation
WBCs: White blood cells
WNT: Wingless-related integration site
