# Supplementary material for: The interplay between cytokine genes and microRNAs in anemia of inflammation among hemodialysis patients
Source: Sci Rep. 2026 May 18;16:15334. doi: 10.1038/s41598-026-49829-w (PMC13184107; doi:10.1038/s41598-026-49829-w)
Supplement: Supplementary file 1 — Supplementary Material 1 [file 41598_2026_49829_MOESM1_ESM.docx]

**Supplementary Table 1.** Pearson correlation study of IL-6, TNF- α, miR-34, miR-130 & miR-16b.

|  | | **miR-34** | **miR-130** | **miR-16b** |
| --- | --- | --- | --- | --- |
| **IL-6** | Pearson Correlation | 0.963** | -0.979** | -0.981** |
|  | *p*. value | <001 | <001 | <001 |
| **TNF-α** | Pearson Correlation | 0.981** | -0.976** | -0.992** |
|  | *p*. value | <001 | <001 | <001 |
| **miR-34** | Pearson Correlation |  | -0.972** | -0.967** |
|  | *p*. value |  | <001 | <001 |
| **miR-130** | Pearson Correlation | -0.972** |  | 0.973** |
|  | *p*. value | <001 |  | <001 |
| **miR-16b** | Pearson Correlation | -0.967** | 0.973** |  |
|  | *p*. value | <001 | <001 |  |

The data were analyzed by Pearson Correlation. * *p* value <0.05 is significant, ** *p* value <0.01 is highly significant.

**Supplementary Table 2.** Prognostic significance of miR-34, miR-130 & miR-16b in HD with AI.

| Test Result Variable(s) | OR | 95%C.I | | p. value |
| --- | --- | --- | --- | --- |
|  |  | Lower | Upper |  |
| miR-34 | 3.143 | 0.896 | 6.547 | <0.001** |
| miR-130 | 0.144 | 0.025 | 0.867 | <0.001** |
| miR-16b | 0.125 | 0.048 | 0.769 | <0.001** |

OR; Odd Ratio, C.I; Confidence Interval,

* *p* value <0.05 is significant, ** *p* value <0.01 is highly significant.

Hosmer–Lemeshow goodness-of-fit test statistic, the Nagelkerke R², and the –2 Log Likelihood to fully characterize model performance.

**Supplementary Table 3.** Correlation coefficients, p-values, FDR (Benjamini-Hochberg adjusted), and sample sizes for the association between each miRNA and the cytokines IL6 and TNF.

|  | **Query** | **Signal Strength statistics** | **P-value** | **FDR (BH)** | **Event_SD** | **Event_TD** |
| --- | --- | --- | --- | --- | --- | --- |
| **miR-16b** | IL6 | 0.2929329025858 | 2.03249252334044e-06 | 9.37890170597992e-05 | 254 | 253 |
|  | TNF | 0.135094609068425 | 0.031372928810086 | 0.118529794843752 | 254 | 252 |
| **miR-34** | IL6 | 0.188807226191744 | 0.00251549084527918 | 0.0131664792013791 | 177 | 253 |
|  | TNF | -0.0260116640792713 | 0.679912341006167 | 0.800462194781044 | 177 | 252 |
| **miR-130** | IL6 | 0.339329566526333 | 2.90954669982753e-08 | 5.52016360166711e-07 | 254 | 253 |
|  | TNF | 0.0479288833969864 | 0.446937718516284 | 0.561483341080071 | 254 | 252 |

*Query – Gene/Site/Protein in given target dataset (dataset with association was performed).*

*Signal Strength – Estimate/coefficient/Statistic obtained from respective statistical method used for analysis.*

*P-value – P-value obtained from statistical method.*

*FDR (BH) – FDR is calculated by BH (Benjamini-Hochberg method).*

*Event_SD – Number of observations in search dataset attribute without NA’s and Zero’s.*

*Event_TD - Number of observations in target dataset attribute without NA’s and Zero’s.*


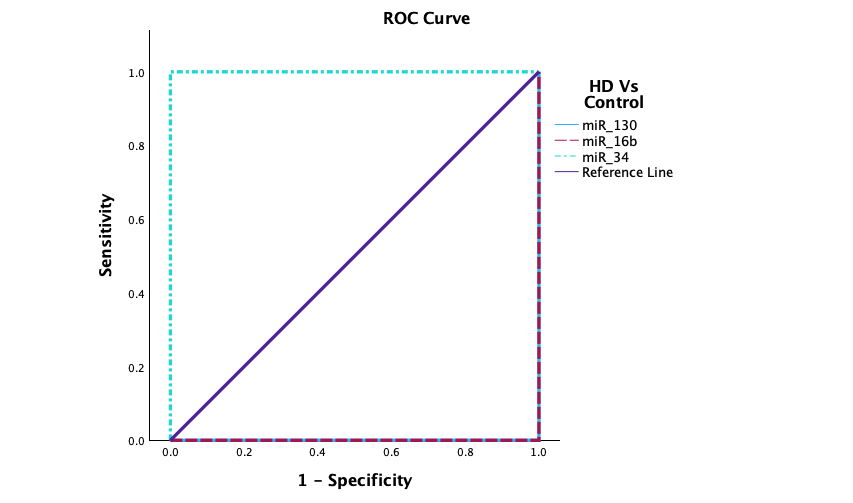


**Supplementary Figure 1.** ROC curve of miR-34, miR-130 & miR-16b in HD with AI.
